# Advances in hair growth

**DOI:** 10.12703/r/11-1

**Published:** 2022-01-12

**Authors:** Dmitri Wall, Nekma Meah, Nicole Fagan, Katherine York, Rodney Sinclair

**Affiliations:** 1 National and International Skin Registry Solutions (NISR), Charles Institute of Dermatology, University College Dublin, Dublin, Ireland; 2 Hair Restoration Blackrock; Dublin, Ireland; 3 Charles Institute of Dermatology, School of Medicine, University College Dublin, Dublin, Ireland; 4 St Helens & Knowsley NHS Trust, Prescot, UK; 5 Manchester University, Faculty of Biology, Medicine and Health, Oxford Road, Manchester, UK; 6 St. James’s Hospital, Dublin, Ireland; 7 Netcare Greenacres Hospital, Port Elizabeth, South Africa; 8 Sinclair Dermatology, Melbourne, Australia

**Keywords:** Alopecia, hair growth, androgenetic alopecia, male pattern hair loss, female pattern hair loss, antiandrogens, minoxidil, platelet rich plasma, prostaglandins, exosomes, low-level laser light, fractional lasers, micro-needling, hair cycling

## Abstract

Hair is a deeply rooted component of identity and culture. Recent articles in this series have focused on scientific evidence relating to hair growth and new insights into the pathogenesis and mechanism of hair loss. This article reviews emerging evidence that has advanced our understanding of hair growth in both of these areas to provide a context for outlining current and emerging therapies. These include finasteride, minoxidil, topical prostaglandins, natural supplements, microneedling, low-level laser light, platelet-rich plasma, fractional lasers, cellular therapy, Wnt activators and SFRP1 antagonism.

## Introduction

A deeply rooted component of identity and culture, the role of hair extends far beyond function, while hair disorders can significantly impact wellbeing and quality of life^1^. One example is in patients with breast cancer, where chemotherapy-induced anagen effluvium has been reported to be “psychologically more difficult than the loss of a breast”^2–4^. Another example is alopecia areata, where suicide has been described^5^. Androgenetic alopecia (AGA) is characterised by patterned hair loss in both men (male pattern hair loss, or MPHL) and women (female pattern hair loss, or FPHL)^6–8^. While the incidence of AGA varies across races, its prevalence increases with age, visibly affecting 57% of women and 73.5% of men who are at least 80 years old^9^.

The frequency and impact of hair loss continue to fuel a search for greater understanding of hair growth and resulting developments in therapeutics. This article aims to build on previous articles in this series that provide an excellent overview of the scientific evidence relating to hair growth^10^ and new insights into the pathogenesis and mechanism of hair loss^11^. We will also endeavour to outline current and emerging therapies to promote hair growth.

## Hair cycling

The hair follicle is a “complex miniorgan” that produces hair shafts from terminally differentiated keratinocytes^12^. In association with the sebaceous gland, apocrine gland and arrector pili muscle (APM), the hair follicle forms the pilosebaceous unit. Eccrine glands have also recently been shown to be integrated within the pilosebaceous unit^13,14^. Scalp hair follicles cluster to form compound pilosebaceous units, consisting of one primary follicle and one or more secondary follicles associated with a single APM and single sebaceous gland^11^. All the hairs from a follicular unit emerge through a shared infundibulum^11^.

On average, a human has between 2 and 5 million hair follicles, of which 100,000 are on the scalp. Blonde-haired Caucasians typically have a higher hair density than dark-haired Caucasians, who in turn have a higher hair density than red-haired Caucasians, Africans and Asians^15–18^. The hair follicle is a complex structure that integrates tissues that arise embryologically from ectoderm, neuroectoderm and mesoderm. Epidermal and mesenchymal cells integrate, proliferate, differentiate and cycle through phases of the hair cycle called anagen, catagen and telogen. The hair fibre forms and elongates during anagen, is retained during catagen and early telogen and then is shed mid-telogen at a point in time called exogen. The late-telogen period after exogen and before the onset of the next anagen, where the follicle is empty, is called kenogen^12,19–22^. As the rate of linear hair growth remains relatively constant throughout life, the main determinant of hair length is anagen duration. Kenogen duration has an impact on hair density but not length.

Hair cycling involves remodelling of the lower “bulb” portion of the hair follicle during catagen. The non-cycling, upper portion of the hair follicle contains the isthmus and infundibulum, which are separated by the sebaceous gland duct. The hair bulge sits on the outer root sheath (ORS) at the lowest point of isthmus, where the APM inserts. The inner root sheath provides a mechanical barrier between the isthmus and the outside world and provides a protective microenvironment for the bulge. The bulge is a stem cell niche for hair follicle keratinocytes and melanocytes as well as arrector pili myocytes^23,24^. Bulge keratinocyte stem cells activate at the transition from telogen to anagen and promote regeneration of the bulb. Transient amplifying cells rapidly proliferate, migrate downwards into the dermis, reconnect with the dermal papilla (DP) to reform the hair bulb and differentiate into matrix cells. Hair bulb matrix cells are rapidly dividing cells that give rise to ORS, inner root sheath, cuticle, cortex and medulla of the hair shaft during anagen. Anagen can continue uninterrupted for many years before ending abruptly at the onset of catagen. Catagen, characterised by rapid apoptotic destruction of the entire hair bulb and partial separation from the DP, is complete in less than 2 weeks. The hair follicle remnant consists of the bulge, isthmus and infundibulum and contains a club fibre with no inner root sheath. Exogen exposes the isthmus to the environment. During telogen, the bulb and papilla remain connected by a fibrous tract. Cross-signalling between the papilla and bulge at the onset of anagen induces the new bulb to migrate down the fibrous tract into the same position as its ancestral bulb^25–27^.

**Figure 1.**
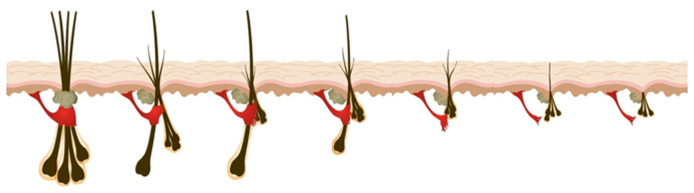
Miniaturisation of the hair follicle. In a previous F1000 article, a model of androgenetic alopecia is presented^11^. Through consecutive hair cycles, progressive miniaturisation of the hair follicle unit occurs, initially affecting secondary follicles, associated with hair density reduction, before the arrector pili muscle is replaced by fat^32^. Ultimately, detachment of this muscle from the regenerative bulge area is associated with irreversible hair loss^11,32^. This figure was adapted from Sinclair *et al*.^11^ which is licensed under Creative Commons Attribution 4.0 International (CC BY 4.0) (https://creativecommons.org/licenses/by-nc/4.0/).

**Figure 2.**
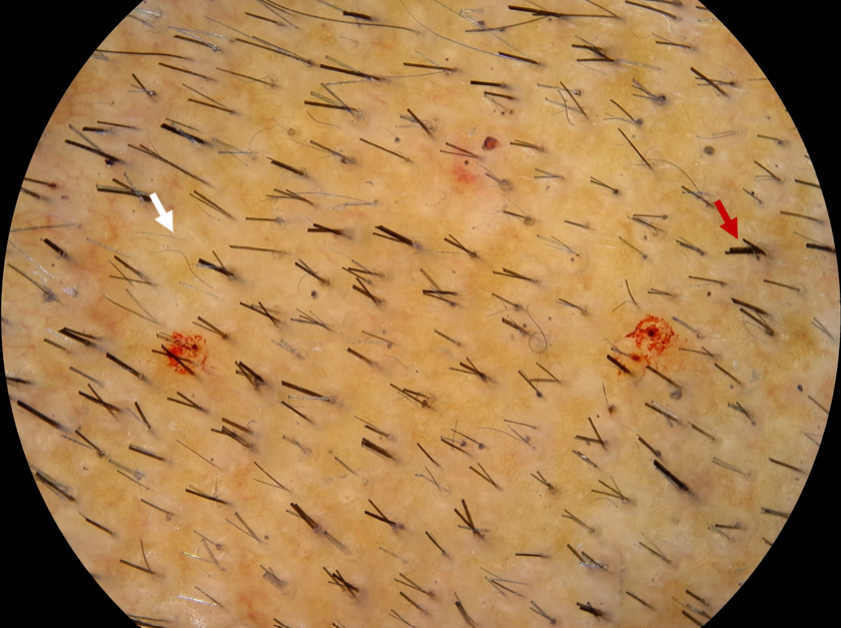
Androgenetic alopecia miniaturised follicles. In androgenetic alopecia, there is a reduction in the number of hairs per follicular unit (white arrow) versus in the normal hair follicular unit (red arrow), where multiple hairs emerge from a single infundibulum. This image was kindly provided by RS from his clinic.

The DP also undergoes dynamic changes during hair cycling. At the onset of catagen, DP fibroblasts migrate into the dermal sheath that surrounds the ORS of the hair follicle and act as a smooth muscle. The dermal sheath, which becomes contractile during catagen, pushes the hair follicle and the surviving DP upwards, enabling its relocation to the upper reticular dermis, just below the bulge^27^. At the onset of anagen, dermal sheath cells migrate into the DP. Dynamic changes in hair follicle size and hair fibre diameter are a consequence of the relative influx/efflux of DP cells into the dermal sheath during hair cycling. The development of secondary sexual hair at puberty involves net recruitment of dermal sheath cells into the DP with each hair cycle. The development of AGA involves the net loss of DP cells into the dermal sheath with each hair cycle (Figure 1), leading to miniaturisation of the hair follicle (Figure 1 and Figure 2).

## Regulation of the hair cycle

Regulation of the hair cycle involves multiple, incompletely understood endocrine, autocrine and paracrine signalling pathways in complex interplay. Of particular note is the Wnt family, and specific roles of members of this family are still to be elaborated. Beta-catenin is a core factor of the Wnt signalling pathway and an essential enabler of stem cell differentiation into follicular keratinocytes^28^. Anagen-to-telogen transition is induced by transient β-catenin signalling^29,30^ and is influenced by cyclical expression of bone morphogenic proteins (BMP2 and 4), produced by dermal fibroblasts and subcutaneous adipocytes^31^, by fibroblast growth factors (FGF 7 and 10), BMP inhibitors (transforming growth factor b2 [TGF-b2] and noggin) and Wnt7b^31,33–36^. Additionally, adipocyte precursor cells express platelet-derived growth factor (PDGF) alpha to activate the PDGF receptor in the DP, resulting in hair germ activation^37^. Perifollicular vascularisation has been shown to increase during anagen and regress during catagen and is related to ORS keratinocyte vascular endothelial growth factor (VEGF) mRNA over- and under-expression respectively^38^. Hepatocyte growth factor/scatter factor has been shown to stimulate hair follicle growth *in vitro*^39,40^, and insulin-like growth factor 1 (IGF-1) has been identified as a significant regulator in the hair follicle^41,42^.

Over the previous decade, a therapeutically relevant new method of paracrine signalling has been identified by means of nanosized extracellular phospholipid bilayer membranous vesicles (exosomes 30–120 nm and microvesicles 100–1000 nm) that can transport lipids, metabolites, nucleic acids and proteins^43–45^. Zhou *et al*. reported that DP cell-exosomes, isolated from healthy human scalp specimens, accelerated the onset of anagen and delayed catagen in mouse models, resulting in increased levels of β-catenin and Sonic hedgehog (Shh)^46^. A recent study identified that human dermal papillae exposed to activated human dermal fibroblasts (hDFs) produce stimulated extracellular vesicles (st-EVs) that secrete the non-Wnt ligand Norrin^43^. It is hypothesised that subsequent activation of the β-catenin pathway is enhanced by hDF-provided Frizzled-4 (Fzd4), the specific receptor for Norrin, resulting in the identified enhanced hair follicle growth *ex vivo*^43^.

Beyond signalling, lymphatic vessels (LVs) have been shown to localise adjacent to the bulge, with increased density and absence respectively in transgenic mice associated with prolonged anagen or accelerated entry into catagen^47^. Furthermore, LV-promoting VEGF-C injections have been shown to promote hair follicle growth in mice.

## Therapeutics

The aim of therapeutics is to safely and effectively harness the mechanisms outlined above, to promote anagen, delay catagen and ultimately prevent or reverse AGA miniaturisation with a view to restoring or maintaining visible hair density. Finasteride and topical minoxidil are the most widely recognised agents for the treatment of AGA. Both are approved by the US Food and Drug Administration (FDA) and the European Medicines Authority^48^. These and other treatments which promote hair regrowth and target specific regulators involved in hair growth will be discussed below.

### Finasteride

Finasteride competitively inhibits the 5-alpha-reductase type 2 enzyme, thus preventing conversion of testosterone to dihydrotestosterone (DHT)^49^. Finasteride is a potential teratogen and generally is contraindicated in women of childbearing potential while the efficacy of topical therapy is limited by poor adherence to treatment. Response varies: some achieve significant regrowth whereas others benefit most from slowing of additional hair loss^50^.

A meta-analysis of placebo-controlled randomised trials identified moderate-quality evidence supporting the use of finasteride for AGA in men^51^. At the end of 48 months of treatment, the mean percentage change in hair count was 24% higher in patients receiving finasteride. Most studies reporting on the benefit of finasteride have focused on the scalp vertex, but importantly a randomised trial of 326 men found it was efficacious for frontal scalp hair thinning as well^52^. It may occasionally adversely affect sexual function, and a systematic review of nine trials, totalling 3570 patients, identified an absolute increase of 1.5% in the risk of erectile dysfunction^51^. These effects usually resolve after discontinuation of the medication^53,54 ^; however, a small percentage have reported continued symptoms for years after stopping the medication^55^. In this regard, a single-centre survey study using a sexual experience scale comparing 99 non-finasteride and 663 finasteride users identified no significant difference in reporting of sexual dysfunction^56^.

More recently, a pharmacovigilance case–non-case study, analysing the World Health Organization’s VigiBase individual case safety report database, showed signals of suicidality and psychological adverse events associated with the use of finasteride in men under the age of 46; however, sensitivity analyses suggested that this may be influenced by “stimulated reporting”^57^.

The side effects associated with systemic finasteride may limit its long-term use. This has led to increased interest in topical finasteride, which is not currently FDA-approved, as an alternative therapy. The safety and efficacy of topical finasteride were first investigated over 20 years ago in a single-blind, placebo-controlled study over a 16-month period^58^. Patient outcomes were favourable, and 73% of patients in the treated group reported “high effectiveness”. Recently, a review exploring topical finasteride in male AGA and FPHL identified that many studies out of the 33 analysed showed positive results and a good safety profile^59^. Recent reviews of the use of topical finasteride in the treatment of AGA suggest that although topical finasteride is a promising therapy that may be non-inferior to systemic finasteride, there is a paucity of evidence-based research^48^. Studies included in the reviews showed decreased hair loss, reduction in balding areas, and increased hair diameter, follicular density, and total hair count. Combination therapy of topical finasteride with other oral and topical agents, such as minoxidil, may have synergistic effects. Although studies showed that DHT levels may be altered, no sexual side effects were noted as a result. Adverse events, such as contact dermatitis, irritation, pruritus and dryness, were due mainly to topical application but were well tolerated. Further research, however, was advised to examine the efficacy of topical finasteride in long-term regrowth, tolerability and side effect profile as well as to assess the optimum topical delivery method in monotherapy and combination therapy.

### Minoxidil

Minoxidil is a 2,4-di-amino-6-piperidinopyrimidine-3-oxide and works in AGA by prolonging anagen, shortening telogen and enlarging miniaturised follicles^60^. Although its mechanism of action has not yet been fully elucidated, transcriptome and proteome analysis has demonstrated differential upregulation of genes in vertex versus occipital scalp of patients with AGA and has demonstrated alteration in expression following treatment with minoxidil^61–64^. It has been shown to increase paracrine growth factor release from and motility of adipose-derived stem cells (ADSCs), enhancing hair growth in mice^65^.

Minoxidil is converted to its active form, minoxidil sulphate, by sulfotransferase enzymes in the hair follicle ORS^66–68^. Studies from the previous decade have demonstrated the potential to predict response to minoxidil on the basis of sulfotransferase activity in plucked hair follicles^66,69,70^. Interestingly, low-dose aspirin has been shown to inhibit hair follicle sulfotransferase activity, possibly impacting on response to topical minoxidil^71^. About 30 to 40% of patients will experience visible regrowth with 6 months of twice-daily application of 5% topical minoxidil^66,72,73^. Early reports from one double-blind study^74^ suggest that increasing the concentration of topical minoxidil, which is not commercially available, to 15% can achieve a clinically significant response in 60% of non-responders to 5% topical minoxidil^74,75^.

The efficacy of minoxidil lotion, however, is limited by poor solubility. Solutions greater than 5% are unstable. Percutaneous absorption is saturated after twice-daily application. Crystallisation (or powdering) of minoxidil occurs on the scalp when the solvent evaporates. Powdering leads to a loss of active substance and also has an undesirable cosmetic effect on the hair. Co-solubilising agents used to prevent powdering further limit usability as they make the hair gummy, sticky or greasy. Usability is especially a problem for women with FPHL who have long hair. Whereas the hair-promoting effect of topical minoxidil generally takes 6 months to become apparent, the median duration of use is 6 weeks. Continuous use is required to maintain the hair-promoting effects of topical minoxidil, yet many who initiate treatment will discontinue use beyond 12 months.

Oral minoxidil initially was developed in the 1950s to treat hypertension. Hypertrichosis was identified as an unwanted side effect but prompted redevelopment as a treatment for AGA as Regaine/Rogaine in the 1980s. Owing to dose-related side effects, namely postural hypotension, fluid retention and hypertrichosis, it has not been used routinely in standard doses for AGA treatment^76^. Its efficacy and safety were evaluated in a 2015 study of 30 men with AGA given 5 mg daily for 24 weeks^77^. Hair growth and total hair count significantly increased in the vertex area, but hypertrichosis and pedal oedema were still common side effects. A recent study on treatment of FPHL involved therapy with 0.25 mg minoxidil combined with 25 mg spironolactone to reduce the risk of fluid retention and augment therapy with its antiandrogen effect^76^. This dose was well tolerated by the majority, and most noticed a reduction in hair shedding at 3 months and increased density at 6 months^76^. A randomised, open, 24-week study comparing oral minoxidil 1 mg with topical minoxidil 5% solution in 52 patients with FPHL identified oral minoxidil as safe and well tolerated, and there was a reduction in shedding superior to topical minoxidil^78^. Although there was no significant difference in hair density, there was a trend towards greater improvement in the oral group. Further data from a large multicentre study have demonstrated its efficacy and safety in AGA^79^.

### Topical prostaglandins

Several studies have highlighted the important role of prostaglandins (PGs) in governing the hair cycle^80^. Prostaglandin D_2_ (PGD_2_) has hair growth–inhibitory effects and is present in increased levels in the AGA-affected scalp, whereas prostaglandin E_2_ (PGE_2_), present in reduced levels in AGA scalps, and prostaglandin F_2a_ (PGF_2a_) have hair growth–stimulating effects^81,82^. A 2019 study in which 32 Hispanic patients with AGA were biopsied demonstrated that men with early MPHL overexpressed the enzyme prostaglandin E synthase (PTGES), which synthesises PGE_2_, suggesting that PGs may play differing roles, depending on the stage of AGA^83^.

Bimatoprost, a synthetic PGF_2a_ analogue^84^, has been demonstrated to increase the thickness, length and darkness of eyelashes^85^ and the fullness and darkness of eyebrow hair^86–90^ by increasing the number of hairs and their duration in the anagen phase^91–93^. To determine the efficacy and safety of bimatoprost scalp solution on scalp hair growth in mild to moderate AGA, a number of clinical trials have been performed^94–96^ and they indicate that topical bimatoprost is not superior in efficacy to topical minoxidil. Latanoprost, a PGF_2a_ analogue, can induce anagen and hypertrophic changes in hair follicles^97,98^. A randomised double-blind placebo-controlled pilot study^99^ assessing 0.1% topical latanoprost daily in 16 men with AGA found that, compared with placebo-treated areas and baseline, hair density increased significantly^99,100^.

### Topical cetirizine

Cetirizine is a second-generation H1 blocker. It has anti-inflammatory properties and decreases production of PGD_2_, which, unlike other PGs, is thought to play a negative role in hair growth. It is safe and has a lower potential side effect profile than other topical therapies, potentially resulting in improved compliance compared with treatments such as topical minoxidil and finasteride. One case-controlled study of 60 patients with AGA showed significantly higher hair regrowth and patient satisfaction in the group that received 1% topical cetirizine (n = 30) than in the control group^101^. In a pilot study of 67 patients who received 1% topical cetirizine, there were increases in total and terminal hair density as well as diameter^102^. Currently, a number of clinical trials are looking at the use of topical cetirizine in AGA, including in females^103^, and comparing 1% topical cetirizine with 5% minoxidil^104^.

### Natural ingredients

Analysis of hair has revealed a composition of iron, oxygen, hydrogen, nitrogen and sulphur. Thus, an adequate supply of blood containing these minerals is essential for hair growth during anagen. Anagen is associated with a rearrangement of skin vasculature, an increase in skin perfusion, and angiogenesis^105^. Various vitamins and minerals are responsible for the modulation of angiogenesis during anagen and therefore are important. In addition, multiple vitamins, minerals, and herbal drugs stimulate hair growth or prevent hair loss by various mechanisms (Table 1)^106–108^ and thus deficiencies in these can cause alopecia. Supplementation with these, in theory, should improve hair growth and this is particularly true for iron deficiency.

**Table 1.** Vitamins, minerals and herbal drugs that stimulate hair growth or prevent hair loss by various mechanisms^106–108^.

**Agents improving blood supply to the scalp:**
Niacin (vitamin B_3_) Vitamin B complex Ascorbic acid (vitamin C) Tocopherol (vitamin E) Grape seed Rosemary oil Sage Nettles Hibiscus rosasinensis^108^
**Cofactors for carboxylases that catalyse an essential step in intermediate metabolism:**
Biotin
**Antioxidants:**
Zinc and grape seed
**Agents that inhibit or reduce 5 alpha reductase activity:**
Inhibitors: green tea^107^, ginkgo biloba and emu oil
**Agents improving hair texture and thus preventing loss of dry brittle hairs:**
Essential fatty acids (primrose and salmon oil) Amino acids (l-cysteine and l-methionine)

### Microneedling

Microneedling is the process of using a roller device apparatus of small fine needles to micro-puncture the stratum corneum of the epidermis. Although the procedure alone can stimulate neovascularisation, growth factor activity and Wnt protein expression^109^, it is often used in AGA in conjunction with hair growth stimulants: minoxidil, plasma-rich protein or topical steroids. When combined, microneedling can facilitate the percutaneous delivery of topical therapies. Lee *et al*. conducted a split-scalp study in 11 women with FPHL^110^. Microneedling was performed on half the scalp treated with growth factors and the other half treated with normal saline. At 5 weeks, the microneedling with growth factor–treated scalp had an increase in hair count (52.91 ± 10.85) compared with the microneedling with saline-treated scalp (45.91 ± 9.98) (*P* = 0.0001).

### Low-level laser

Low-level laser therapy (LLLT) is occasionally synonymous with red light therapy, cold laser and soft laser^111^. It is thought to exert a biomodulation/biostimulation effect on tissue, promoting anti-inflammatory effects^111–115^. The exact mechanism of action in stimulating hair regrowth is not known. Improved cellular activity, reduced inflammation and improved microcirculation may be involved^108^. The therapeutic effects are delivered in wavelengths ranging from 500 to 1100 nm (red to near infrared) at low energy density (3 to 90 mW/cm^2^)^111,116^. A variety of LLLT devices, including in-salon hoods or overhead panels, are available to patients with hair loss. Bonnets, helmets or hand-held combs are suitable for home use. Both the HairMax^®^ LaserComb and the head cap TOPHAT 665 have had FDA clearance for treatment in AGA.

Kim *et al*. conducted a 24-week, double-blind randomised controlled trial (RCT) comparing sham device to LLLT helmets (emitting wavelengths of 630-nm, 650-nm and 660-nm light-emitting diodes) in 40 patients with AGA^117^. The mean hair density was significantly greater in the LLLT group versus the sham group. A 26-week RCT involving 269 patients with AGA produced similar results. Patients were randomly assigned to receive different models of the HairMax^®^ LaserComb (7-beam, 9-beam, 12-beam and 9- and 12-beam) or a sham device. The mean terminal hair count at 26 weeks from baseline was higher in the LaserComb subjects compared with the sham-treated subjects. The LaserComb patients also reported a higher percentage of overall improvement with their hair (with respect to hair thickness and fullness) compared with sham-treated subjects^118^. However, in an earlier case series of seven patients receiving twice-weekly LLLT, the findings were not conclusive. Although increases in terminal hairs and in hair shaft diameter were noted, the findings were not statistically significant and global image assessment did not support an improvement with LLLT^119^. Liu *et al*. conducted a system review and meta-analysis of RCTs, reviewing eight studies with 11 double-blind RCTs, and concluded that LLLT resulted in a significant increase in hair density and that low-frequency treatment can result in a better effect than high, and types of devices and course duration did not impact the effectiveness on hair growth^120^.

### Platelet-rich plasma

Treatment with platelet-rich plasma (PRP) involves intradermal injection of the scalp with a concentrated volume of platelets, within a small volume of plasma, that has been derived from the centrifugation of a patient’s own venous blood^121,122^. PRP contains numerous PDGFs, including PDGF, TGF-b1 and TGF-b2, VEGF, basic fibroblast growth factor, endothelial growth factor and insulin-like growth factors^121–124^. It is proposed that PRP prolongs anagen, prevents catagen and shortens the period from telogen to anagen through the release of growth factors that stimulate “cell survival, proliferation, and differentiation”^125–128^.

A mechanistic model from Gupta and Carviel describes the stimulation of hair growth with key roles played by “growth factor mediated increased activation of wingless (Wnt)/β-catenin, extracellular signal-regulated kinase (ERK), and protein kinase B (Akt) signalling pathways”^127^, to improve vascularisation^129^ and prolong anagen^127^. A meta-analysis of six studies and 177 patients demonstrated increased hair number per square centimetre after PRP versus control in addition to a significantly increased hair thickness cross-section per 10^−4^ mm^2^ in the PRP group^122^. Earlier studies demonstrated greater improvement in hair thickness when combined with additional therapies. Combination therapy of PRP and polydeoxyribonucleotide (PDRN), delivered over 12 sessions, yielded a greater improvement in hair thickness in subjects with FPHL than treatment with PDRN therapy alone^130^. The largest study, involving 64 patients with AGA, is by Schiavone *et al.*^131^ Patients received leukocyte PRP plus concentrated plasmatic proteins at baseline and at 3 months. Using macrophotographs and Jaeschke rating of clinical change, two unblinded assessors noted some improvement in 62 patients. The mean changes in clinical rating were 3.2 (95% confidence interval [CI] 2.9–3.5) and 3.9 (95% CI 3.5–4.3).

In a double-blind, controlled study of 26 patients randomly assigned to receive four injections of saline or PRP, those receiving the latter were found to have significant increases in hair density, count and percentage of anagen hairs^132^. Interestingly, there was no correlation with either platelet counts or measured growth factors, including epidermal growth factor, VEGF or PDGF, prompting the authors to speculate whether other, unmeasured growth factors are related to the measured response to treatment. Clinical practice often involves introduction of PRP in combination with other therapies. In a retrospective review, a significant increase in hair density, but not calibre, was seen in 17 out of 24 patients 2 months after initial injections^133^. All 24 patients were already using 5% topical minoxidil, while 20 patients were also taking an oral antiandrogen.

A meta-analysis of 10 studies (n = 165 participants) examining PRP treatment compared with baseline calculated a statistically significant overall standardised mean difference in PRP-treated patients of 0.58^134^. Six of the studies (n = 99 participants) compared PRP with placebo, and the PRP group showed greater efficacy (standardised mean difference of 0.51). This led the authors to conclude that PRP treatment is beneficial in AGA. Another systematic review and network meta-analysis comparing relative efficacy of 2% minoxidil, 5% minoxidil, finasteride, PRP and LLLT indicated that LLLT had a greater increase in mean difference in hair count compared with the other treatments, which were nearly equivalent, although the quality of evidence, based on risk-of-bias assessment, was noted to be very low^135^. Although meta-analysis highlights evidence of increases in hair number and thickness with PRP in small studies, it is important to recognise that these studies lack comparability, highlighting an unmet need for larger RCTs^122,136^.

### Fractional lasers

Fractional lasers are indicated for the treatment of rhytides and scarring; however, the role of lasers in treating alopecia is relatively new. Ablative (fractional 10,600-nm carbon dioxide and fractional 2940-nm erbium: yttrium aluminium garnet [YAG]) and nonablative (fractional 1550-nm erbium glass) lasers have been investigated in the context of alopecia areata and AGA^124,137^. Fractional lasers are unique in creating pixelated microthermal injury zones (sparing the epidermis and dermis), allowing better tolerability and fewer cutaneous side effects^137^.

In a study involving 28 patients with FPHL, patients received 10 treatments with 1550-nm fractional erbium glass laser at 2-week intervals^138^. Improvement in hair density and mean hair thickness was observed after 5 months of treatment; mean percentage changes from baseline were 57% and 77% respectively. Global photographs also confirmed improvement in 24 patients^138^. Furthermore, Kim *et al*. investigated “the effects of a 1,550 -nm fractional erbium-glass laser on the hair cycle in an alopecia areata mouse model” by laser irradiating the shaved backs of C3H/HeN mice whose hair was in telogen stage. Molecular studies revealed an increase in Wnt 5a and β-catenin signal levels, while histopathologic studies demonstrated conversion from telogen to anagen conversion. Following these results, the authors conducted a pilot study of 20 patients with MPHL treated with 1550-nm fractional erbium glass laser (five sessions at 2-weekly intervals) and found improvements in hair density and growth rates^139^.

### Hair Stimulating Complex

Hair Stimulating Complex™ (HSC), patented by Histogen (San Diego, CA, USA)^140^, comes as an injectable, cell-conditioned media made up of keratinocyte growth factor, VEGF and follistatin. These growth factors are involved in stem cell proliferation and stimulate hair growth. In an RCT of 26 subjects receiving HSC, significant improvement in hair growth was noted over placebo at 12 and 52 weeks^141^. HSC-treated areas had increased hair shaft thickness (6.3% ± 2.5% vs. −0.63% ± 2.1%; *P* = 0.046), thickness density (12.8% ± 4.5% vs. −0.2% ± 2.9%; *P* = 0.028) and terminal hair density (20.6 ± 4.9% vs. 4.4 ± 4.9%; *P* = 0.029).

### Cellular therapy

***Autologous dermal sheath/dermal papilla/epidermal cells.*** Hair follicle regeneration is initiated by mesenchymal DP cell signalling to stem cells in the bulge region of the hair follicle. A viable pool of functional DP cells is necessary to maintain this process long-term. In healthy follicles, dermal sheath cup (DSC) cells may help to repopulate DP cells for each regenerative hair cycle. A gradual decline in DP numbers may account for AGA^142,143^. Research has shown that transplanted DP and DSC cells can promote DP formation and hair follicle induction^144^. Therefore, DP/DSC cells harvested from androgen-resistant areas, expanded in culture and then injected into balding scalp potentially stimulate hair growth. Several phase II trials have attempted to explore the efficacy of autologous dermal cells and/or epidermal cells injected into balding scalp to reverse miniaturisation in AGA^145,146^. Although study statuses are confirmed as complete, results are outstanding and not yet published.

RepliCel (Vancouver, BC, Canada) have conducted a randomised, double-blind, placebo-controlled phase I/IIa trial to evaluate the safety and efficacy of intradermal injection of human autologous hair follicle DSC cells in 10 men and 9 women with AGA^147^. Stabilisation of hair loss was seen in all per-protocol participants at 24 months. Of those participants who achieved greater than a 5% hair density increase over baseline at 6 months, the top ten were noted to benefit from a sustained response (average 4.2%) at 24 months^148^. Over a 5-year follow-up period, no serious adverse events were reported.

***Adipose-derived stem cells.*** Mesenchymal stem cells, rich in adipose tissue (ADSCs)^149^, are multipotent cells that influence surrounding cells through the generation of growth factors^150,151^ and have demonstrated a capacity to promote hair growth *in vitro* and *in vivo*^151–154^. ADSCs are available as a prepared conditioned media or extracted from adipose tissue using liposuction. This is administered to the balding scalp by serial injections. A pilot case series has suggested a possible role for autologous stem cell–enriched fat grafting for the treatment of AGA^155^. In an observational study of 27 patients with FPHL, improved hair density was observed in patients who received commercially available ADSC-conditioned media^151^. Stromal vascular fraction, containing adipose tissue–derived mesenchymal stem cells alone^155^ and in combination with PRP, has demonstrated significant benefit in a small number of AGA patients in one study^156^ and superior results compared with PRP alone in a further study^157^. Stem cell–conditioned media, rich in paracrine factors, has also been investigated as an alternative to cell-based therapy^158,159^.

***Issues relating to cellular and regenerative therapies.*** Although advances in cellular and regenerative therapies for hair growth continue to evolve, many regulatory and ethical challenges exist. These include problems with activation of stem cells, which are dependent on pathways that may be lost in an environment altered by AGA^160^. As demonstrated by the use of stem cell–conditioned media as a therapy in hair loss^158,159^, it is becoming increasingly clear that the environment that the stem cell is exposed to is equally as important as the cell itself^160^. Indeed, both the cellular and acellular components of the physical environment of the stem cell are important and complicate *in vitro* analysis and the prediction of behaviour in a degrading *in vivo* state. Therefore, the ability to model the stem cell niche *in vitro* and predict efficacy *in vivo* remains a largely unmet need and is a significant challenge in the study of regenerative medicine in hair therapy.

Concerns exist that evidence generated regarding cellular and regenerative therapies can be over-hyped or unreproducible or have insufficient power to reasonably address safety concerns. Financial gain motivating the provision of unregulated services with safety concerns or little evidence must also be considered^161^. A report published in 2020 by the European Academies’ Science Advisory Council and the Federation of European Academies of Medicine identified significant ethical and regulatory challenges associated with regenerative medicine^162^. The report highlights how the ethical concerns of regenerative medicine, like those of many other fields of cellular research, typically relate to safety and efficacy, patient consent, misleading information, professional responsibility, equity and fairness as well as issues surrounding donated biological material^162^. Although there are strict regulatory frameworks for clinical experimental studies, there can often be premature marketing of therapies, facilitated by regulatory bodies promoting initiatives such as rapid and accelerated approval. Cossu *et al*. urge that regulatory procedures for regenerative medicine be transparent, robust, evidence-based, rapid and accurate and argue that much work still needs to be done, including engaging with the public, to counter misinformation^163^.

### Wnt activators – Valproic acid

Lee *et al*. demonstrated hair regrowth with topical application of valproic acid to C3H mice^164^. Unlike minoxidil, valproic acid was found to activate the Wnt/β-catenin pathway. Other inducers of the Wnt/β-catenin pathway, including 4-phenyl butyric acid (PBA), lithium chloride and beryllium chloride, were also investigated and found to stimulate hair regrowth *in vivo*. Treatment with lithium chloride or beryllium chloride, in particular, triggered anagen entry. In an RCT of patients with AGA treated with topical valproic acid (VPA) or placebo for 24 weeks^165^, the mean change in total hair count was found to be significantly higher in the VPA group than in the placebo group (*P* = 0.047). Both groups experienced mild adverse effects.

### Miscellaneous

Newer therapies have recently emerged. Microarray analysis led to the discovery of downregulation of secreted frizzled-related protein 1 (*SFRP1*), a Wnt inhibitor, by cyclosporine A^166^. In hair follicles, *SFRP1* regulates Wnt/β-catenin activity. Using an SFRP1 antagonist (WAY-316606), the authors were able to demonstrate enhanced hair regrowth *ex vivo*. Sildenafil, a phosphodiesterase 5 (PDE5) inhibitor, has been shown to promote hair growth in mice. Sildenafil was found to stimulate growth factor expression (VEGF and PDGF), upregulate ERK phosphorylation and promote angiogenesis. Finally, 7-phloroeckol, a metabolite of a brown marine algae, has been shown to stimulate DP cells and ORS cells *in vitro*, inducing IGF-1 and promoting hair growth in human hair follicles *in vitro*^167^.

## Conclusions

Treatment of AGA remains a challenge and patients with AGA often have a heterogenous response to treatment, partly because the complex aetiopathogenesis, particularly in affected women, has not yet been fully elucidated. Intricate pathways regulate hair cycling and anagen duration and determine hair growth. As we discover more about these pathways, we move into an era of a growing number of potential therapeutics for hair growth promotion. A number of ongoing clinical trials are exploring novel treatments; however, it is unlikely that one therapy alone will result in a desired, sustainable outcome. Combination therapy incorporating systemic therapy and adjuvant procedural modalities (PRP, LLLT or fractional laser) may well represent the optimal strategy to produce long-lasting results, prior to surgical considerations.

## Abbreviations

ADSC, adipose-derived stem cell; AGA, androgenetic alopecia; Akt, protein kinase B; APM, arrector pili muscle; BMP, bone morphogenic protein; CI, confidence interval; DHT, dihydrotestosterone; DP, dermal papilla; DSC, dermal sheath cup; ERK, extracellular signal-regulated kinase; FDA, US Food and Drug Administration; FPHL, female pattern hair loss; hDF, (activated) human dermal fibroblast; HSC, Hair Stimulating Complex; IGF-1, insulin-like growth factor 1; LLLT, low-level laser therapy; LV, lymphatic vessel; MPHL, male pattern hair loss; ORS, outer root sheath; PDGF, platelet-derived growth factor; PDRN, polydeoxyribonucleotide; PG, prostaglandin; PGD_2_, prostaglandin D_2_; PGE_2_, prostaglandin E_2_; PGF_2a_, prostaglandin F_2a_; PRP, platelet-rich plasma; RCT, randomised controlled trial; SFRP1, secreted frizzled-related protein 1; TGF, transforming growth factor; TGF-b1, transforming growth factor b1; TGF-b2, transforming growth factor b2; VEGF, vascular endothelial growth factor; VPA, topical valproic acid
